# Introduction of a pyramid guiding process for general musculoskeletal physical rehabilitation

**DOI:** 10.1186/1746-1340-14-9

**Published:** 2006-06-08

**Authors:** Timothy W Stark

**Affiliations:** 1 Health Sciences Division, School of Chiropractic, Murdoch University. South Street, Murdoch, Western Australia, Australia

## Abstract

Successful instruction of a complicated subject as Physical Rehabilitation demands organization. To understand principles and processes of such a field demands a hierarchy of steps to achieve the intended outcome.

This paper is intended to be an introduction to a proposed pyramid scheme of general physical rehabilitation principles. The purpose of the pyramid scheme is to allow for a greater understanding for the student and patient. As the respected Food Guide Pyramid accomplishes, the student will further appreciate and apply supported physical rehabilitation principles and the patient will understand that there is a progressive method to their functional healing process.

## Background

Musculo-skeletal dysfunction requiring physical rehabilitation can be quite diverse in cause, severity, chronicity, complicating factors, location of injury, and the anatomy involved. Because of the multifactorial involvement of musculo-skeletal dysfunction, it can be a challenge knowing when and where to start the physical rehabilitation process – not to mention teaching this process to students in the fields of chiropractic, medicine, physical/physio and occupational therapies.

After a thorough physical assessment of an acute or chronic musculo-skeletal dysfunction (or injury), the clinician may determine a need for progressing the patient into a physical rehabilitation program. The first issue is to avoid inducing any more harm to the patient than what has already occurred. This element of safety involves the implementation of correct diagnosis, timely rehabilitation intervention, correct rehabilitation program design, and correct progression within the program [1].

The pyramid introduced in this paper will assist the clinician by adding further safety and guidance to physical rehabilitation implementation. This paper will introduce a "general" pyramid for guidance that can be adapted to most of the regions of the body. Future publications from this author will include modified pyramid guides for physical assessment and rehabilitation application for specific regions of the human body.

As a musculo-skeletal clinician and lecturer in the areas of sports injury care and physical rehabilitation, the author finds this pyramid to be beneficial in instructing patients on their process of rehabilitation and is extremely helpful when teaching physical rehabilitation principles to students.

Like all 1^st ^edition publications, this pyramid will evolve as others in the field provide input. The author looks forward to this evolution process and participating in further discussions on this topic.

### History of Pyramids

Pyramids are used by professions across the health care scheme. Some of the first health care professions to use the pyramid as a guide were the nutritionists or dieticians. The Food Guide Pyramid expresses to the lay-person and practitioner the emphasis on specific foods (such as grains) and the need to limit other food types (such as simple sugars). These food guide pyramids are now replicated across the world to meet the diverse cultures; Mediterranean, Asian, Latin, Puerto Rican, Vegetarian, Soul Food [2], Japanese, and Native Hawaiians [3]. Advancements in the utilization of this guide have been expanded to include weight management by using the Food Pyramid Score where positive or negative points are earned when specific servings are eaten according to the tiers of the pyramid. [4]

The Fitness Professionals also boast of a well-designed pyramid expressing their concern for limiting sedentary lifestyles and focusing on plenty of lifestyle physical activities [5].

**Figure 1:**
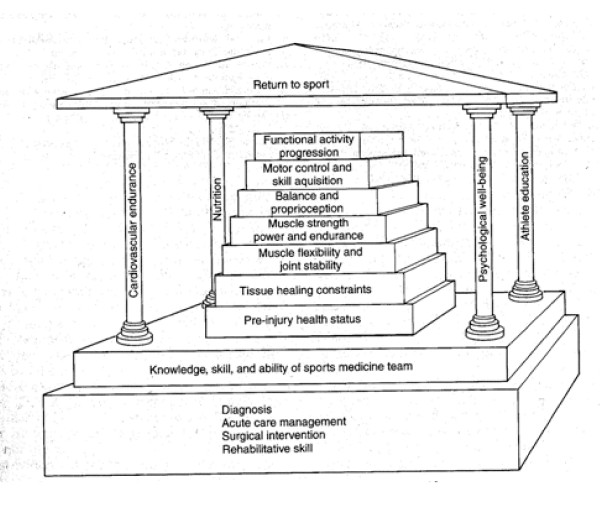
Sports Rehabilitation Pyramid.

A complicated Sports Rehabilitation Pyramid was published quite some time ago, Fig. 1 (reference not found...). However, based on current knowledge of engrams [6], as well as general strength and conditioning principles, the picture is likely no longer appropriate [7].

This sports rehabilitation pyramid/structure (Figure 1) is more thorough for the sports medicine concept, including elements beyond physical rehabilitation. However, with the rehabilitation components, there should be greater emphasis on balance and proprioception training (regional stability) prior to muscle strength, power, and endurance [7]. It's the author's opinion that strength and power training of a body region prior to acquiring optimum motor control and joint stability places the patient at risk and is inefficient.

O'Connor, et al. described another, five step management pyramid in the field of sports medicine and rehabilitation that included 1.) control of inflammation, 2.) promote healing, 3.) increase fitness, 4.) control abuse, and 5.) return to activity.

Although, not in a pyramid format, Hyde and Gengenbach nicely noted four phases of rehabilitation and appropriately encouraged progressing the rehabilitation process from phase I to phase IV [7]. This author has some similarities to their process but does describe a number of differences.

The various pyramids and concepts differ for certain reasons that exceed the interest of this article, but the important point is that the pyramid framework of educating the lay-person and practitioner is easy to understand and appears to be internationally accepted.

The proposed pyramid that this paper will formally introduce defines tiers of specific physical rehabilitation progression, consistent with the categories of the 2^nd ^and 3^rd ^steps of O'Connor's article. It is expected that the treating physician and/or therapist will have determined that a patient is beyond the acute phases of inflammation control and that, based on objective data, they are a good candidate for non-surgical care, including physical rehabilitation.

### The proposed physical rehabilitation Pyramid

#### Explanation and rationale for the individual mutli-tiered system

**Figure 2:**
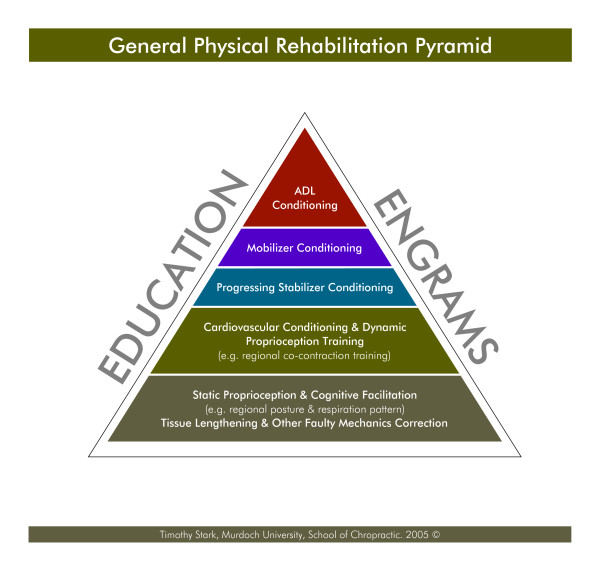
The proposed Physical Rehabilitation Pyramid.

Similar to other pyramid schemes, the bottom tier should be considered the first and most important and implemented before moving to the next tier, (figure 2). Each patient must be evaluated for these components and the clinician must be satisfied that moving to the next tier will not hinder the patient's healing and rehabilitation process. Further details will be explained in the following paragraphs.

#### Education and engrams

Throughout the physical rehabilitation process, it should be understood that the patient must be educated appropriately by the clinician. It is this author's experience that an educated patient is an inspired and compliant patient. If the patient understands that there are desired goals to meet before progressing to the next tier, the patient may be more focused on their in-clinic and home assignments in order to reach their goals and progress. Additionally, the clinician needs to monitor how the patient naturally moves and performs their exercises; looking for muscle substitution (such as using excessive trapezius contraction for glenohumeral abduction), asymmetry in movement (such as demonstrating greater hip extension with one hip v. the other during gait), and/or sub-optimal regional function (such as diminished core stability during a squatting manoeuvre). If these aberrant movements are occurring, the patient should not progress to the next tier and the patient should be educated about these findings so they may be able to apply the conscious changes during the exercises and also during activities of daily living.

#### Tier 1 (bottom tier): static proprioception, tissue lengthening, and other faulty mechanics correction

This tier has the greatest importance. One might have appropriate motor patterns or inappropriate motor patterns. Motor patterns require a great degree of muscle coordination that may either be under conscious or unconscious control. When a clinician wishes to train a motor pattern, it will require numerous conscious attempts before this motor pattern becomes an unconscious pattern, or engram [8] If the patient is demonstrating a poor motor pattern in a static state, i.e. poor posture (rounded shoulders), would this aberrant pattern, or engram, be further enforced if allowed to progress throughout repetitive dynamic activities, such as cardiovascular conditioning? If so, progressing to the next tier may re-enforce this poor engram [9]. Additionally, tissues that are in an unwanted shortened state may affect the static and dynamic proprioception and engram of the patient by modifying the patient's posture and motion [10]. When reviewing literature for musculo-skeletal rehabilitation, it was common to find instruction for beginning isometric exercises early to prevent muscle strength loss [11]. However, implementing strengthening exercises of any form (isometric, isotonic, or isokinetic) may place the patient in an inefficient state regarding overall muscle function. Janda [10] stated that pronounced tightness of a muscle group is consistent with a weakened muscle. Implementing strengthening exercise may perpetuate the tightness and develop further weakness. Therefore, strengthening exercises should be postponed, and lengthening procedures, such as Graston Technique, MRT (myofascial release techniques), and stretching should be implemented. Other faulty mechanics may include joint restrictions such as vertebral segmental dysfunction or shoulder capsular-shortening which may require additional therapy such as mobilization or manipulation.

#### Tier 2: Cardiovascular conditioning and dynamic proprioception (regional co-contraction)

It is well appreciated that early intervention of cardiovascular conditioning enhances tissue healing via tissue oxygenation and nutrition; decreases potential for muscle atrophy and physical stress on the newly formed collagen fibres [12]; and has positive effects psychologically [13]. As important as this element of tissue healing is, it is the author's opinion that performing repetitive movements for an extended period of time with poor proprioception – and therefore possible poor coordinated movements – may further encourage poor engram development [9]. This tier also involves improving dynamic proprioception such as a normal gait or normal glenohumeral rhythm. One well accepted method of increasing joint proprioception is co-contraction [14]. A favourite exercise technique for co-contraction, especially of the core and upper extremities, is using oscillatory stabilization such as the Bodyblade^®^. The Bodyblade^® ^is a reactive, oscillating device that utilizes inertia to generate up to 270 muscle contractions per minute [15]. The patient pushes and pulls on the apparatus, which accelerates the blade and creates a force due to the flex or amplitude of the blade. The greater the flex, the greater the resistance that is needed by the body to counteract the destabilizing forces delivered into the body. The blade's movement therefore requires the user to contract his or her muscles in order to neutralize these forces [15]. Also routinely utilized are balance boards, which allow for a natural oscillation and co-contraction and are well accepted to be beneficial for dynamic proprioception [10]. When reviewing the literature, it also seemed to be a common recommendation to implement open-chain exercises (exercise where the distal aspect of the extremity is not in contact with anything, e.g. seated leg extensions) before implementing closed-chain exercises (exercises where the distal extremity is in contact with a surface, e.g. squats) [7]. I prefer implementing closed-chain exercises as early as possible. There appears to be a greater amount of regional co-contraction with exercises [11], and therefore possible benefit to the patient by enhancing dynamic proprioception during the exercise.

#### Tier 3: Progressing stabilizer conditioning

There are numerous philosophies for classifying muscles of a joint: phasic v tonic, and stabilizers v. mobilizers are two examples. Stabilizers are defined as smaller muscles that perform joint stability functions such as joint surface centration [14]. These muscles are generally smaller than the mobilizers, closer to the joint, and tend to be more fatigue-resistant. Based on what we know about stabilizer function it appears that it would be wise to gain (or regain) optimum function of these smaller muscles to improve joint stability before progressing onto mobilizer conditioning. An example of this would be to condition the smaller rotator cuff muscles of the glenohumeral joint before implementing larger and multi-plane strengthening exercises for the mobilizers of the joint such as the deltoids [16]. It has also been demonstrated that performing stabilizer strengthening in end-ranges of motion of a joint is also beneficial to enhancing the stability of a joint [17] Exercises that this author prefers includes continuing with oscillatory stabilization exercises (low resistance, high repetition and small AROM) as described in the prior tier but adding slow and controlled movements; for example, starting the oscillation using the right arm and then slowly moving the shoulder throughout its full pain-free ROM. Muscle endurance and neuromuscular control for joint stability are goals during this tier.

#### Tier 4: Mobilizer conditioning

As discussed previously, the mobilizer muscles may tend to be larger, further from the joint, and may play a role in larger and more powerful joint function [14]. They tend to fatigue earlier than healthy stabilizer muscles. After the clinician feels comfortable that the patient's stabilizer muscles have achieved an appropriate level of conditioning and will aid in protecting and stabilizing the joint, it may be safe and beneficial for the patient to progress to larger and higher intensity conditioning. The intensity and movements should progress towards activities that the patient will be required to perform once discharged. In this tier of rehabilitation, continued endurance as well as strength and power are goals.

#### Tier 5: ADL's

During this tier of the Physical Rehabilitation Pyramid the ultimate goal is to prepare the patient for a safe return to their Activities of Daily Living (ADL). This of course could include laying bricks, child-care, house-work, or playing footy. During this tier's rehabilitation, the clinician will focus on progressing from the 4^th ^tier and assure that the ADL movements are performed with evidence of good engrams, strength, endurance, etc. For athletes, this is an appropriate time to implement special motor skills such as speed and agility [1]. For the non-athlete, it is important to assess and condition for compound movements such as bending at the waist and twisting to lift a two year old child. Current literature suggests [13] that this may be referred to as "functional training" and that such training has led to decreased time off of work and increased speed of returning to sport.

Throughout this process, continually re-assessing, using outcome measurements, is important in order to confirm that the patient is benefiting from care. Lastly, before discharge from this physical rehabilitation program, there is a need for an exit physical examination to confirm tissue integrity, psychological readiness, and appropriate education for decreasing the risk of re-injury [1].

## Discussion

The scope of this paper is to consider the active physical rehabilitation process and the safe and effective progression of these processes. However, acute injuries may require passive care initially for pain management, inflammation control, etc. Such implementation would include PRICE (Protection, Rest, Ice, Compression and Elevation) [13]. Additional therapies such as medication and modalities may also play an important role in this phase of healing (PRICEMM) [1]. Additionally, ruling-out psychological concerns, surgery, and more aggressive interventions should be confirmed by the appropriate physician. And, equally as important, consulting a clinician who is trained in physical rehabilitation will be important before implementing such therapy.

## Conclusion

Physical rehabilitation is a complicated process of musculo-skeletal healing and recovery which should be patient-sensitive and condition-specific (i.e. not every shoulder condition (e.g. rotator cuff injury) can go straight into three sets of ten repetitions of tubing exercises for internal and external rotation). Like a toddler learning to walk for the first time: if they are not strong enough to weight bear or have the necessary static proprioception, they will not be able to stand. If they do not have the motor control and dynamic proprioception to shift weight from one leg to the other, they will not walk. Simply because a toddler can stand does not mean she can safely walk. Each level of physical progression requires added neuromuscular and cognitive ability and conditioning.

Just as the Food Guide Pyramid has progressed over the years, it had to start from something, and this proposed pyramid, just in its infancy, will also progress. If accepted by the rehabilitation community, such progress will include addition of pictures demonstrating actions at each tier (similar to the Food Guide Pyramids), rigorous clinical testing to demonstrate it's effectiveness, and specific pyramids for different regions of the body. This author encourages feedback and discussion on rationale for changes and improvements in this process for enhanced safety and efficacy of patient management and clinician education.

## Competing interests

The author(s) declare that they have no competing interests.
